# Influence of solvents in the preparation of cobalt sulfide for supercapacitors

**DOI:** 10.1098/rsos.170427

**Published:** 2017-09-06

**Authors:** Yedluri Anil Kumar, S. Srinivasa Rao, Dinah Punnoose, Chebrolu Venkata Tulasivarma, Chandu V. V. M. Gopi, Kandasamy Prabakar, Hee-Je Kim

**Affiliations:** School of Electrical Engineering, Pusan National University, Busandaehak-ro 63beon-gil, Geumjeong-gu, Busan 46241, Republic of Korea

**Keywords:** cobalt sulfide, supercapacitors, energy storage devices, cyclic voltammetry, charge–discharge

## Abstract

In this study, cobalt sulfide (CoS) electrodes are synthesized using various solvents such as water, ethanol and a combination of the two via a facile chemical bath deposition method on Ni foam. The crystalline nature, chemical states and surface morphology of the prepared CoS nanoparticles are characterized using X-ray diffraction, X-ray photoelectron spectroscopy, scanning electron microscopy and transition electron microscopy. The electrochemical properties of CoS electrodes are also evaluated using cyclic voltammetry, galvanostatic charge–discharge and electrochemical impedance spectroscopy. When used as an electrode for a supercapacitor, CoS prepared with ethanol as a solvent exhibits a capacitance of 41.36 F g^−1^ at 1.5 A g^−1^, which is significantly better than that prepared using water and water/ethanol-based solvents (31.66 and 18.94 F g^−1^ at 1.5 A g^−1^, respectively). This superior capacitance is attributed to the ideal surface morphology of the solvent, which allows for easy diffusion of electrolyte ions into the inner region of the electrode. High electrical conduction enables a high rate capability. These results suggest that CoS nanoparticles are highly promising for energy storage applications as well as photocatalysis, electrocatalysis, water splitting and solar cells, among others. These results show that CoS is a promising positive electrode material for practical supercapacitors.

## 1. Introduction

Recently, a growing demand for energy resources has necessitated the development of high performance, environmentally friendly components for storage applications and energy generation [1,2]. One of the most promising components for the next generation of power devices is the supercapacitor, or electrochemical capacitor; it has higher energy density than lithium batteries and conventional dielectric capacitors. Thus, supercapacitors are emerging as promising energy storage devices with excellent power density, high energy efficiency, reversibility, durability, fast dynamic charge propagation, sustainability and low cost. As such, this technology has attracted considerable attention in recent years [3,4].

Based on the energy storage mechanism, supercapacitors are divided into three categories: electrical double-layer capacitors (EDLCs), pseudocapacitors and hybrid capacitors [5]. In EDLCs, the charge storage is based on the formation of an electrical double-layer between the electrolyte interface ions and electrode surface [6]. Carbon materials such as carbon nanofibres, carbon nanotubes, activated carbon black, carbon aerogel, graphene oxide, graphite and graphene exhibit EDLC behaviour [7]. EDLC materials have a large surface area and high power density, which are useful for electrochemical energy storage. However, they do have some drawbacks such as limited specific capacitance, high resistance, low energy density and limited rate capability [8].

The electrode materials used in pseudocapacitors rely on the presence of a redox-based fast faradaic reaction at the surface of the electrode and near the intercalation of electrolytes, which exhibit relatively large capacitance values from stored energy. They also have higher energy than EDLCs [9]. While all organic conducting polymers are applicable as electrode materials in supercapacitors, they do have some disadvantages such as a short life cycle, slow transport of kinetic ions and they can undergo corrosion in the presence acidic electrolytes [10].

Therefore, various chemically synthesized pseudocapacitive materials have been evaluated for their potential applicability as supercapacitors. In particular, metal oxides, metal hydroxides and metal sulfides have been extensively investigated owing to their relatively fast redox kinetics and high capacitance [11–13]. The electrochemical performance of pseudocapacitive materials largely depends on their surface area and morphology. Many metal oxides such as MnO_2_, NiO, V_2_O_5,_ Co_3_O_4_ and RuO_2_, as well as hydroxide materials such as Co(OH)_2_ and Ni(OH)_2_, exhibit high capacitance values [14].

The large energy storage capacity of RuO_2_ has been extensively researched owing to its potential to function as a supercapacitive electrode with metallic conductivity and reversible faradaic reactions; however, RuO_2_ has presented some difficulties such as high costs, commercial restrictions and the toxicity of ruthenium [15,16]. Moreover, metal oxides and hydroxides demonstrate a gradual loss of capacitance owing to their high resistance, poor conductivity and sparse electron transport. These weaknesses have hindered their practical applications, rendering them inappropriate for commercialization [17]. Hence, research is in progress to develop new electrode materials with desirable capacitive performance, cost effectiveness and good electrical conductivity [18].

Recently, transition metal sulfides have attracted attention for their potential as pseudocapacitive materials for supercapacitors [19]. Previous studies suggest that metal sulfides are better than metal oxides and metal hydroxides. Therefore, metal sulfides have been used as typical pseudocapacitive materials with a relatively high capacitance. Many researchers have studied the supercapacitive properties of various types of metal sulfides such as copper sulfide, nickel sulfide, nickel cobalt sulfide, zinc sulfide, molybdenum disulfide and cobalt sulfide [20].

Among these, cobalt sulfide (CoS) has been considered as one of the most promising and versatile pseudocapacitive electrode materials for supercapacitors, solar cells, catalysts, lithium ion batteries and magnetic materials. CoS has multiple advantages such as low cost, electroactivity, high theoretical specific capacitance and long-term stability. CoS can occur in various phases, including CoS, CoS_2_, Co_2_S_3,_ Co_9_S_8_ and Co_4_S_3_ [21]. The surface morphology, surface area, particle size and porosity of CoS change its specific capacitance; therefore, its properties can vary dramatically according to the solvent used in its preparation. As such, this pseudocapacitive material is being used and researched extensively for a variety of applications.

In this study, a low cost and facile strategy was developed for the design and fabrication of CoS nanostructure on nickel foam using a chemical bath deposition (CBD) method for supercapacitor application. Different chemical methods such as one-step ion exchange, hydrothermal, reflux and CBD have been endorsed in the past for the preparation of CoS. In this study, we report an inexpensive and facile CBD method that we used to obtain different nanoparticles with which to form CoS thin films deposited on a Ni foam substrate. Nanostructured cobalt sulfide was synthesized using cobalt nitrate hexahydrate as the cobalt source and thiourea as the sulfur source. The resulting CoS was deposited using various solvents, and its performance was tested in an aqueous solution of 1 M KOH. Our results show that CoS exhibited good electrochemical properties for supercapacitor applications. Its specific capacitance of 41.36 F g^−1^ at 1.5 A g^−1^ with ethanol as a solvent was higher than that with water and water/ethanol-based solvents (31.66 and 18.94 F g^−1^ at 1.5 A g^−1^, respectively). These interesting results highlights the potential of CoS supercapacitor as a high performance energy storage system for practical applications.

## 2. Material and experimental methods

### 2.1. Materials

All chemicals, such as cobalt nitrate hexahydrate (Co(NO_3_)_2_·6H_2_O), thiourea (CH_4_N_2_S), potassium hydroxide (KOH), absolute ethanol (C_2_H_6_O) and nickel foam (Ni foam) were purchased from Sigma-Aldrich and used as received.

### 2.2. Preparation of CoS/Ni foam electrodes with different solvents

All reagents used were of analytical grade and were not purified further. The electroactive CoS materials were deposited on Ni foam substrates using a facile CBD method. Before depositing the CoS, the Ni foam was cleaned with acetone, ethanol and deionized water for 10 min each, using ultrasonication. It was then dried using a hair dryer.

CoS solutions were prepared using 1 M Co(NO_3_)_2_·6H_2_O and 1 M CH_4_N_2_S as cobalt and sulfide sources, respectively, in 50 ml of water, ethanol and water/ethanol. The resulting solutions were stirred vigorously for 15 min to obtain homogeneous solutions. Next, the prepared Ni foams were immersed vertically into the solutions and placed in a hot air oven at 90°C for 4 h. After air-cooling to room temperature, the CoS-coated Ni foams were washed several times with water and ethanol to remove unwanted particles. The foams were then dried with a hair dryer and placed in an oven at 60°C for 2 h. CoS was prepared with three different solvents: water, ethanol and water/ethanol.

### 2.3. Characterization and measurements

Films consisting of CoS crystals on the prepared electrodes were characterized via X-ray diffraction (XRD) on a D8 ADVANCE, using a CuKα radiation source operated at 40 kV and 30 mA with 2*θ* ranging from 20–80°. The elemental valance states in CoS were analysed through X-ray photoelectron spectroscopy (XPS; VG Escalab 250) with a hemispherical energy analyser, using monochromatic AlKα radiation. The morphology of the samples was studied using a scanning electron microscope (SEM; SU-70, Hitachi) operated at 10 KV, at the Busan Korea Basic Science Institute (KBSI). The elemental composition of the thin film was characterized using energy-dispersive X-ray spectroscopy (EDX), operated at 10 KV.

The particle size of CoS was examined using transmission electron microscopy (TEM: Jem 2011, Joel corps) at the Busan KBSI. The supercapacitive properties of the CoS thin films were studied at room temperature using cyclic voltammetry (CV) and galvanostatic charge–discharge (GCD) on a CHI 600 electrochemical work station. The CV was conducted in a potential window from −0.4 to +0.6 V using scan rates ranging from 10 to 100 mV s^−1^, while the GCD tests were performed within a potential range of −0.2 to 0.5 V, at a current density of 1.5 to 9 A g^−1^. The specific capacitance, energy and power density were calculated from the GCD curves, according to equations (2.1)–(2.3), where *C*_s_ is the specific capacitance (F g^−1^); Δ*t* is the discharge time difference in seconds; *m* and Δ*V* are the mass of the electrode (mg) and potential difference during the discharge process (V), respectively; *E* is the energy density (W h kg^−1^); *P* is the power density (W kg^−1^).

2.1 $$\begin{array}{r} C_{s}=\frac{i\Delta t}{m\Delta V}, \end{array}$$

2.2 $$\begin{array}{r} E=\frac{1}{2}C_{s}{\left(\Delta V\right)}^{2}\times \frac{1000}{3600} \end{array}$$

2.3 $$\begin{array}{r} \;\text{and}\;\;P=\frac{E}{t}=\frac{i\Delta V}{2m}\times 1000, \end{array}$$

where *i* is the discharge current, *t* is the discharge time, *V* is the potential window and *m* is the mass of the active material.

## 3. Results and discussion

### 3.1. Structure and surface morphology characterization

**Figure 1.**
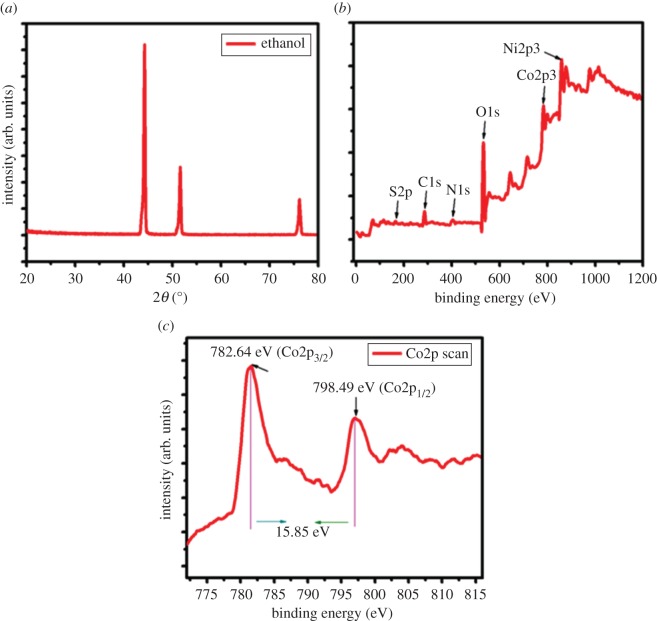
(*a*) XRD pattern of the as-prepared CoS and (*b*,*c*) XPS survey spectrum of CoS using ethanol as solvent and the magnified Co2p peak on the surface of nickel foam substrate.

To identify the crystal structure, we performed XRD and XPS analysis, as shown in figure 1*a–c*. Figure 1*a* clearly shows that there are no prominent or well-defined peaks in the XRD pattern for the CoS. This clearly indicates that the prepared sample is amorphous or that it shows poor crystallinity. This result is consistent with previous reports [22].

The exterior and synthetic states of the Co and S molecules within the prepared CoS were evaluated using XPS, the results of which are shown in figure 1*b*,*c*. We observed different elements such as Co2p3 (781.49), S2p (162.03), C1s (284.6), N1s (399.36), Ni2p3 (856.1), O1s (531.34), P2p (132.52) in the surveyed spectrum of the CoS samples prepared using ethanol as a solvent. Thus, the cooperative affiliation between oxygen, carbon and air within the samples caused a Ni peak, and may be responsible for surface absorption. The XPS spectrum of CoS prepared using ethanol showed two major peaks, corresponding to Co2p_3/2_ and Co2p_1/2,_ at binding energies of 782.64 and 798.49 eV, respectively [23]. The binding energy difference was approximately 15.85 eV. The XPS spectra of S2p are provided in electronic supplementary material, figure S1. These results suggest that CoS was successfully deposited on Ni foam substrates via CBD.

**Figure 2.**
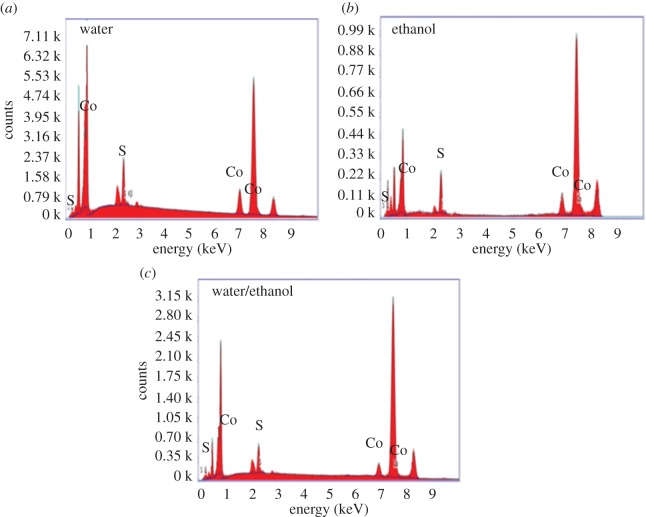
EDX of CoS electrode with different solvents (*a*) water (*b*) ethanol and (*c*) water/ethanol, on Ni foam substrate.

Figure 2 shows the EDS results of CoS prepared using water, ethanol, and water/ethanol as solvents. These parameters are described in table 1. Figure 2 shows the EDS diffraction peaks of Co, S, Ni, O and C within the CoS on Ni foam. Figure 2 confirms the atomic percentage of cobalt and sulfide at 8.14 and 4.68%, respectively, for the water-based electrode. The atomic percentage of Co and S decreased from 8.14 to 4.33% and 4.68 to 2.37%, respectively, when the solvent changed from water to ethanol. Moreover, the percentage of Co decreased and S increased when we used a mixture of water and ethanol (1 : 1) as the solvent. XPS and EDS results suggested that the CoS was successfully deposited on Ni foam substrates.

**Table 1.** Atomic percentage of Co, S, Ni, O and C in various conditions.

condition	at.% of Co	at.% of S	at.% of Ni	at.% of O	at.% of C
water	8.14	4.68	59.7	27.48	—
ethanol	4.33	2.37	50.21	10.15	32.94
water/ethanol	3.67	2.48	66.98	8.32	18.55

**Figure 3.**
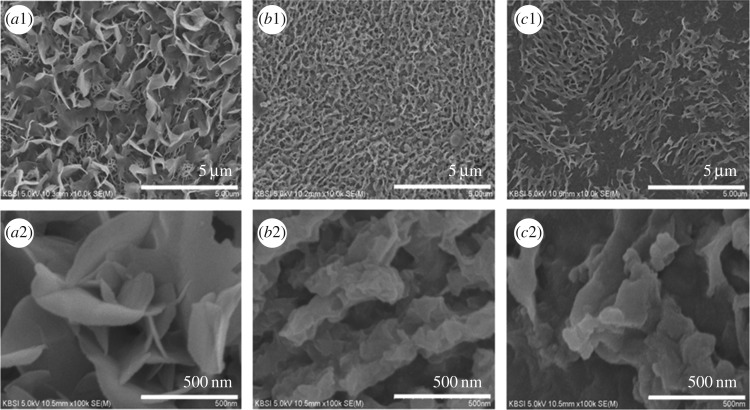
SEM images of the CoS electrode on Ni foam at different magnification in various solvents such as water (*a*1,*a*2), ethanol (*b*1,*b*2) and water/ethanol (*c*1,*c*2).

The surface morphology, size, and surface structure of CoS were analysed via SEM (figure 3). The corresponding electrodes were prepared using the CBD method. The SEM images show a nanoflakes structure, poor adhesion and a small surface area. When CoS was prepared using water as a solvent (figure 3(*a*1,*a*2)), unfledged nanoflakes formed because of a fast increasing rate and high nucleation. When the material had a large pore size and less adhesion to its substrate, it was difficult for ions to diffuse and electrons to be transferred within the electrode. However, this sample also exhibited less adhesion and a large pore size.

Figure 3(*b*1,*b*2) show the SEM images of CoS prepared using ethanol as the solvent. This sample exhibited greater adhesion to its substrate, and had more, but smaller, pores. Moreover, CoS tapioca sago-like nanoparticles prepared using ethanol were distributed uniformly in comparison to CoS prepared using water and water/ethanol. The uniformly coated nanoparticles on the Ni foam showed many electron pathways owing to their relative proximity. Figure 3(*c*1,*c*2) show the SEM images of CoS prepared using water/ethanol. The CoS nanoparticles were dispersed unevenly, with voids between the particles on the surface of the Ni foam. This resulted in poor electrochemical reactivity between the KOH electrolyte and CoS electrode.

**Figure 4.**
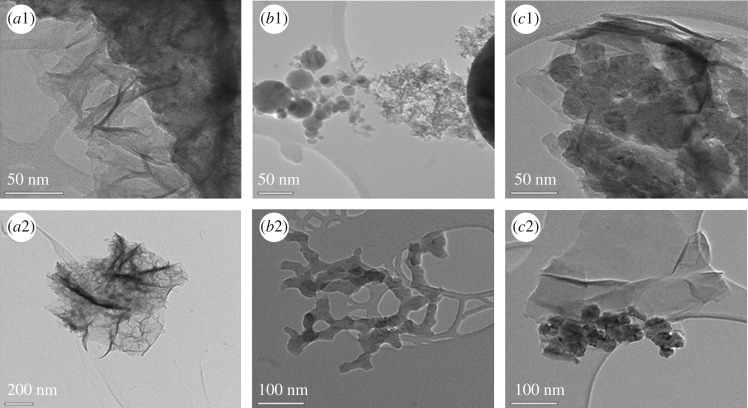
TEM images of the CoS electrode on Ni foam at different magnification in various solvents such as water (*a*1,*a*2), ethanol (*b*1,*b*2) and water/ethanol (*c*1,*c*2).

TEM images (figure 4) were used to further investigate the particle size and structure of CoS prepared with different solvents on Ni foams. It can be seen in figure 4(*b*1,*b*2) that individual nanoparticles form large agglomerations. Moreover, nanoparticles of different sizes have a uniform shape, which can help electrolyte ions penetrate into the inner surface of the CoS electrodes. Previous studies suggested that thin electrode films often outperform other materials because the electrolyte ions and electrons can be more easily transferred than in thicker films. As seen in figure 4(*c*1,*c*2), irregular CoS nanoparticles are formed, which results in a low surface area. From figures 3 and 4, we can conclude that the tapioca sago-like nanoparticles were distributed uniformly. This suggests that CoS electrodes prepared using ethanol have the potential for high capacitive performance.

### 3.2. Electrochemical studies

**Figure 5.**
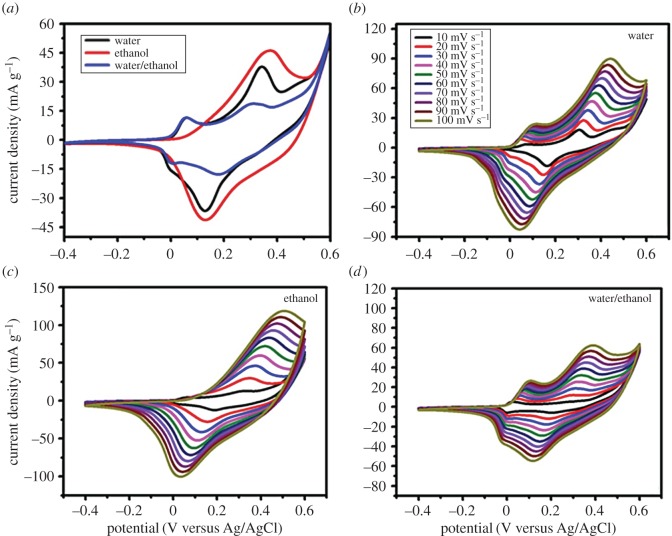
(*a*) A comparison of CV curves of the water, ethanol, water/ethanol; as solvents of the CoS electrodes at the scan rate of 30 mV s^−1^. CV curves of the (*b*) water, (*c*) ethanol and (*d*) water/ethanol at different scan rates (10–100 mV s^−1^) in 1 M KOH solution.

The electrochemical characteristics of CoS electrodes prepared using different solvents were investigated using CV measurements in a three-electrode system. Figure 5*a* displays the CV curves of the CoS electrode in 1 M KOH electrolyte. A potential window of 0.4–0.6 V and a scan rate of 30 mV s^−1^ were used. The oxidation and reduction values for water were 37.80 and −36.85 mA, respectively. Ethanol had values of 45.85 and −41.47 mA, while water/ethanol had values of 18.80 and −17.94 mA, respectively. The CoS electrode prepared using ethanol as a solvent showed the highest oxidation and reduction values among all the electrodes.

Moreover, the surface area of the CV curves for different solvents can be correlated with the SEM and TEM results, as shown in figures 3 and 4. Lower oxidation and reduction values were observed for CoS prepared using water and water/ethanol owing to the resulting large pore size, reduced surface area and poor adhesion to the substrate [24–26]. This led to less electrochemical interaction between the KOH electrolyte and electrode. In addition, a diminished surface area was also observed for the water and water/ethanol-based electrodes owing to the phase segregation of the material [27,28]. Finally, the ethanol-based electrode showed a pair of well-defined redox peaks corresponding to a reversible faradaic reaction. This indicated a superior pseudocapacitance when compared with water and water/ethanol-based films.

Figure 5(*b–d*) shows CV measurements with different scan rates, ranging from 10 to 100 mV s^−1^, for three different CoS electrodes in the same electrolyte. A similar behaviour, for example, two pairs of redox peaks, was observed for all conditions, mainly resulting from pseudocapacitance rather than EDLC. On increasing the scan rate from 10 to 100 mV s^−1^, the anodic and cathodic peaks shifted to a more positive and negative potential, respectively. This may be because of the electrolyte ions being able to access only the outer surface of the electrodes [1,29,30]. At a low scan rate, the electrolyte ions were able to access the inner surface of the electrodes. The oxidation and reduction values gradually increased as scan rate increased. For better understanding, the oxidation and reduction values with respect to scan rate are given in electronic supplementary material, table S1. From the CV results, the prepared CoS electrode exhibited good reversibility and an excellent rate capability when ethanol was used as a solvent.

**Figure 6.**
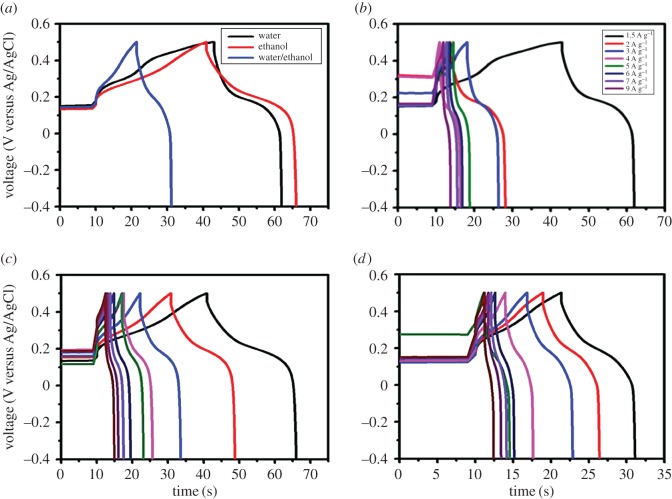
Galvanostatic charge and discharge curves of the (*a*) A comparison of various solvents such as water, ethanol, water/ethanol at the current density of 1.5 A g^−1^, (*b*) water, (*c*) ethanol and (*d*) water/ethanol; as a solvents of CoS electrode at different current densities (1.5–9 A g^−1^) in 1 M KOH solution on Ni foam.

The GCD curves for the CoS electrodes are shown in figure 6. The performances of CoS electrodes prepared using different solvents were compared at a constant current density of 1.5 A g^−1^ and potential range between 0.5 and −0.2 V. These measurements were conducted using 1 M KOH electrolyte (figure 6*a*). CoS electrodes prepared with different solvents had a specific capacitance of 31.66 F g^−1^ (water), 41.36 F g^−1^ (ethanol) and 21.7 F g^−1^ (water/ethanol) at a current density of 1.5 A g^−1^. This excellent capacitive performance was attributed to their tapioca sago-like structure, which allowed the KOH electrolyte to easily diffuse into the inner region of the CoS electrode. This facilitated fast electron transport with a high rate capability, which was consistent with the SEM images.

The three electrodes were further tested at various current densities ranging from 1.5 to 9 A g^−1^, as shown in figure 6(*b–d*). The pseudocapacitance decreased gradually in response to an increase in the current density, owing to insufficient faradaic redox reactions [31–34]. This may have been because at lower current densities, the electrolyte had sufficient time to access the electrode material, which was not possible under a high current density because the reaction rate was high. The specific capacitance values for different electrodes with different current densities are discussed in more detail in table 2.

**Table 2.** Specific capacitance of CoS prepared with different solvents such as water, ethanol and water/ethanol; at different current densities from 1.5 to 9 A g^−1^.

	water	ethanol	water/ethanol
current density (A g^−1^)	specific capacitance (F g^−1^)	specific capacitance (F g^−1^)	specific capacitance (F g^−1^)
1.5	31.66	41.1	24.51
2	32.22	39.68	21.70
3	27.4	37.56	20.2
4	24.71	35.82	18.3
5	23.61	32.5	18.94
6	22.46	30.2	16.8
7	21.15	29.08	16.02
8	18.5	26.75	14.93
9	22.58	25.2	13.33

Ultimately, CoS electrodes prepared with ethanol exhibited higher specific capacitance, electrical conductivity, energy density and greater surface area when compared with electrodes prepared using water and water/ethanol. Continued progress toward optimizing the concentrations of materials, as well as their deposition time, temperature, fabrication techniques and surface morphology, will enable greater specific capacitance for supercapacitors.

A CoS tapioca sago-like nanostructured film was prepared with various solvents and successfully deposited on a Ni foam substrate using as a CBD method. CoS prepared with ethanol as the solvent exhibited multiple advantages over other samples such as a higher utilization rate of electrodes in KOH solution, easy penetration of OH^−1^ ions into the inner region of the electrode and improved faradaic reactions with electrolytes on the electrode surface in comparison to other samples. These results indicate that the CoS electrode can be a good candidate for use in supercapacitors.

**Figure 7.**
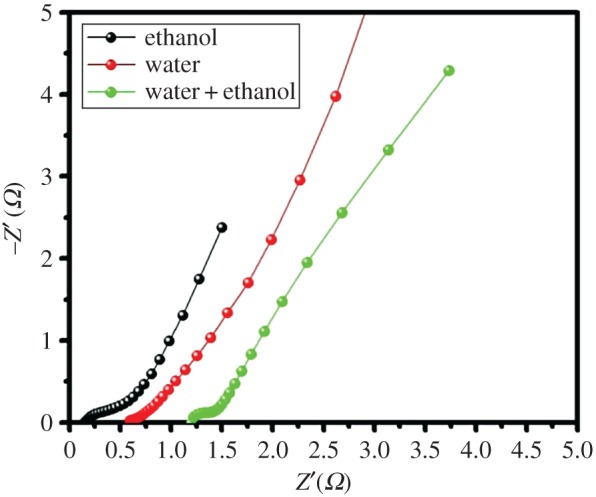
Impedance Nyquist plots of CoS electrode with different solvents ethanol, water and water/ethanol.

The EIS measurements were further carried out to investigate the electrochemical behaviour of various solvents of the CoS electrode. Figure 7 shows the Nyquist plots of the various electrodes over the frequency range of 100 mHz to 500 kHz. Figure 7 depicts that the ethanol solvent of CoS electrode exhibited a small semicircle in the high frequency range and more vertical line in the low frequency range than the water and water/ethanol as solvents, implying good capacitive behaviour with the ion diffusion transport and interfacial charge transfer resistance. These results demonstrated that the combination of electron transfer and fast ion diffusion resulted in remarkable electrochemical performance of the ethanol as a solvent of CoS electrode.

**Figure 8.**
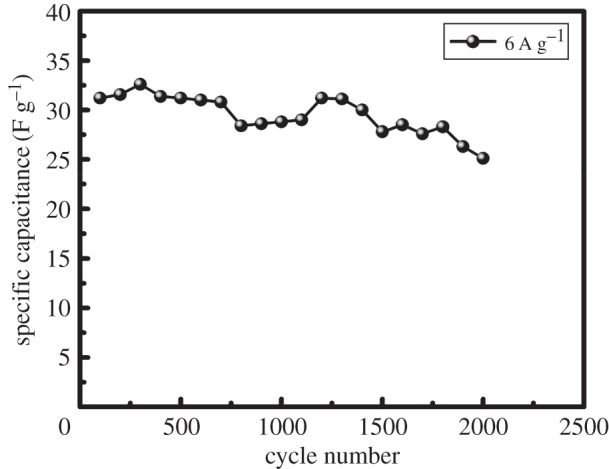
Cycling performance of the ethanol-based CoS electrode at a high current density of 6 A g^−1^ for 2000 cycles.

The long-term cycling stability of an electrode is also most important for practical supercapacitor applications. Figure 8 shows electrochemical stability of CoS electrode with a solvent of ethanol by GCD at a current density of 6 A g^−1^ for 2000 cycles. For the ethanol solvent of the CoS electrode the real capacitance is 31.2 F g^−1^ in the first cycle, and it gradually decreases to 25.1 F g^−1^ after 2000 cycles, showing 19.2% loss after 2000 cycles, indicating the good cycle property as a good electrode material for electrochemical supercapacitor. These positive results are mainly due to with the electrolyte gradually penetrating into the electrode, more and more electrode materials become activated and contribute to the increase of specific capacitance.

## 4. Conclusion

The optimized CoS electrode prepared using ethanol yielded interconnected nanoparticles on a Ni foam substrate. This suggests that the electrode could function well in a supercapacitor owing to its good specific capacitance of 41.36 F g^−1^ at 1.5 A g^−1^, as well as excellent conductivity and rate capability in comparison to electrodes prepared with water and water/ethanol (31.66 and 18.94 F g^−1^ at 1.5 A g^−1^, respectively). This outstanding performance can be attributed to its unique tapioca sago nanostructure constructed by interlaced nanoparticles. Our future work will focus on improving the cycling stability of the CoS electrode. The overall improved electrochemical performance of the CoS electrode prepared using this simple CBD method makes it a promising material for potential applications in high performance supercapacitors because of its low cost and simple preparation and environmental friendliness.

## Supplementary Material

Influence of solvents in the preparation of cobalt sulfide for supercapacitors

## Supplementary Material

Supporting Information

## Supplementary Material

Supplemenatary information

## Data accessibility

We have conducted our experiment systematically and reported their experimental procedure clearly in the experimental section and provided all necessary data in Results and discussion section in the main manuscript.
