# Supplementary material for: Influence of solvents in the preparation of cobalt sulfide for supercapacitors
Source: R Soc Open Sci. 2017 Sep 6;4(9):170427. doi: 10.1098/rsos.170427 (PMC5627093; doi:10.1098/rsos.170427)
Supplement: Influence of solvents in the preparation of cobalt sulfide for supercapacitors [file rsos170427supp1.docx]

**Supporting Information**

**Influence of solvents in the preparation of cobalt sulfide for supercapacitors**

**Anil Kumar Yedluri, S. Srinivasa Rao, Dinah Punnoose, Chebrolu Venkata Tulasivarma, Chandu V. V. M. Gopi, Kandasamy Prabakar and Hee-Je Kim^*^**

^a^ School of Electrical Engineering, Pusan National University, Busandaehak-ro 63beon-gil, Geumjeong-gu, Busan, 46241, Rep. of KOREA

^*^Corresponding Author. Tel: +82 51 510 2364. Fax: +82 51 513 0212.

E-mail: [heeje@pusan.ac.kr](mailto:heeje@pusan.ac.kr)

(H.-J. Kim).

**
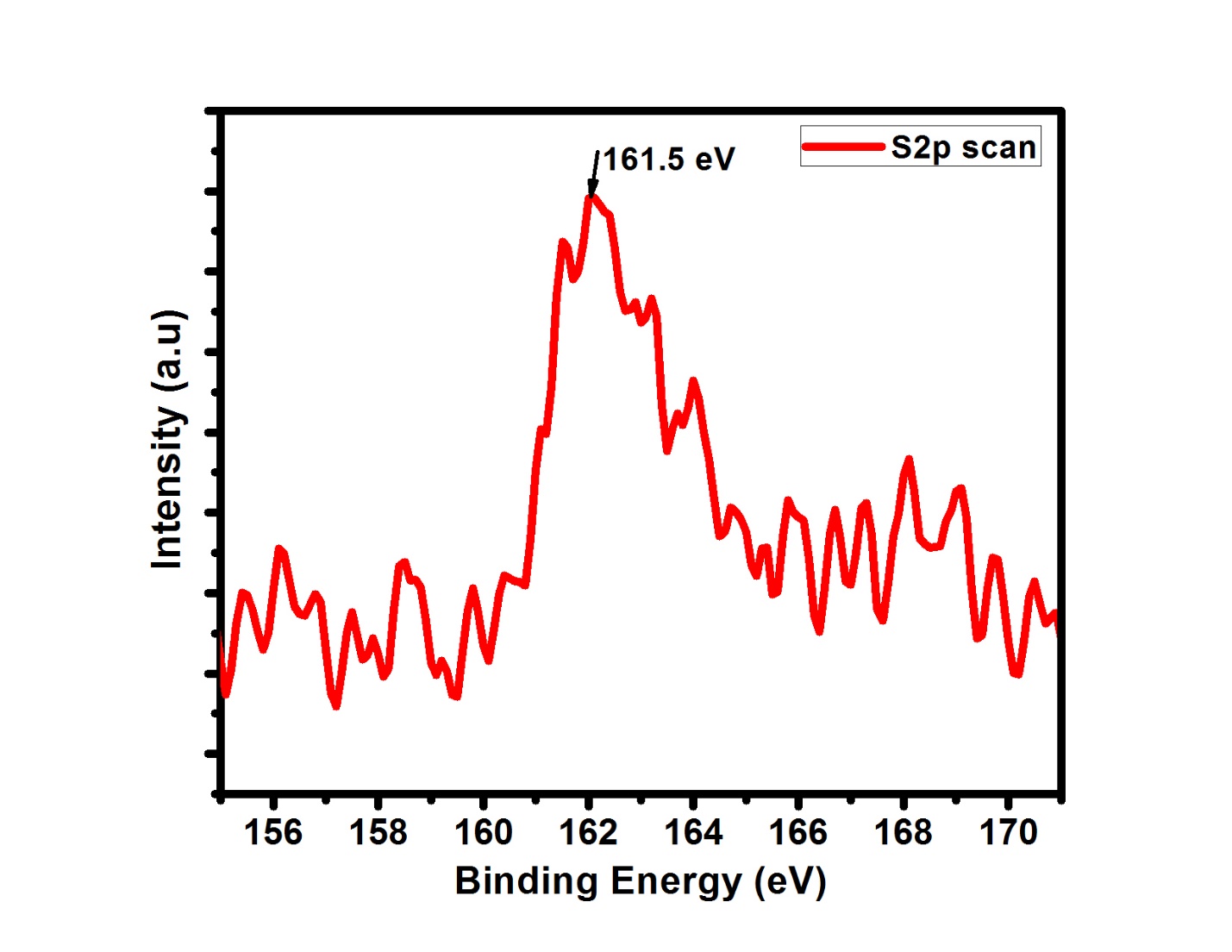
**

**Fig. S1** XPS spectrum of S2p prepared using ethanol as solvent.

| **Scan Rate** | **Water** | | **Ethanol** | | **Water:ethanol** | | |
| --- | --- | --- | --- | --- | --- | --- | --- |
|  | **Oxidation**  **Current (mA)** | **Reduction**  **Current (mA)** | **Oxidation**  **Current (mA)** | **Reduction**  **Current (mA)** | **Oxidation**  **Current** | **Reduction**  **Current** | |
| 10 | 18.35 | -18.34 | 22.30 | -20.81 | 5.10 | -6.03 |  |
| 20 | 27.40 | -27.35 | 29.90 | -27.95 | 12.18 | -11.91 |  |
| 30 | 37.80 | -36.85 | 45.85 | -41.47 | 18.80 | -17.94 |  |
| 40 | 45.10 | -43.19 | 59.66 | -52.29 | 25.63 | -23.60 |  |
| 50 | 54.94 | -52.29 | 72.53 | -62.58 | 32.02 | -29.26 |  |
| 60 | 63.14 | -59.34 | 82.79 | -71.45 | 38.96 | -35.12 |  |
| 70 | 70.46 | -65.94 | 93.05 | -79.49 | 45.28 | -40.01 |  |
| 80 | 77.16 | -71.15 | 102.36 | -87.36 | 51.34 | -44.80 |  |
| 90 | 83.81 | -77.13 | 110.54 | -92.86 | 56.90 | 49.70 |  |
| 100 | 89.97 | -82.52 | 118.48 | -100.17 | 62.42 | -54.63 |  |

**Table S1** Oxidation and reduction current densities of CoS prepared with different solvents such as water, ethanol and water:ethanol at different scan rate from 10-100 mV/s.
